# Can genomic research make a useful contribution to social policy?

**DOI:** 10.1098/rsos.220873

**Published:** 2022-11-23

**Authors:** Kathryn Asbury, Tom McBride, Rosie Bawn

**Affiliations:** ^1^ Department of Education, University of York, York YO10 5DD, UK; ^2^ Ending Youth Violence Lab, Behavioural Insights Team, London SW1H 9NP, UK; ^3^ University of Exeter, Stocker Road, Exeter EX4 4PY, UK

**Keywords:** behavioural genomics, social policy, genetic literacy, DNA, ancestral diversity, policy-making

## Abstract

As genetic research into outcomes beyond health gathers pace, largely through the use of genome-wide association studies, interest from policy-makers has grown. In the last year, two UK reports have explored the policy implications of genomic research, one from the UK Government Office for Science and one from the Early Intervention Foundation. In this article, we explore areas of consensus between these two reports and use them to propose priorities for policy-makers as we prepare for what some have termed a ‘genetic revolution’. Both reports agree on two clear recommendations for science and policy communities. One of these relates to public education and engagement, and the other to ensuring that genomic databases are ancestrally diverse. Both reports agree that—even if it is found to be a viable and ethical idea in the medium-term future—DNA data should not be incorporated into social policy before these two issues have been comprehensively addressed. In the article, we argue that scientists are taking the lead on tackling the diversity deficit but that there is a clear role for policy-makers to play in addressing low genetic literacy in society, and that this is a matter of urgency.

## 1. Introduction

Genome-wide association studies (GWAS) are gathering pace in identifying specific genetic variants associated with outcomes such as educational attainment, income and neurodivergence (e.g. [1–4]). This leaves policy-makers around the world facing a scientific, social, practical and ethical conundrum in terms of whether and how to use this research in a policy context.

In the last year, two UK reports have explored the policy implications of genomic research. The first of these was published by the Early Intervention Foundation (EIF) in August 2021 [5], and the second by the Government Office for Science (GO Science) in January 2022 [6]. It is worth noting that two of the current authors (K.A. and T.M.) were also authors of the EIF report, and that K.A. was an expert reviewer for the GO Science report. In this article, we discuss the motivations underpinning the production of each of these reports, along with their key findings and recommendations. The GO Science report explored potential future contributions of genomic research to policy focused on employment, sport, criminal justice and synthetic biology but, in this review, we focus on social policies relating to education, and child and adolescent development. We consider the key findings and recommendations from both reports in relation to each other and to relevant academic literature.

It is clear that the role genes play in explaining individual differences, the role these differences play in influencing important outcomes (e.g. educational success, income and mental health) and the ethical dilemmas this creates for individuals, societies and governments is receiving increased public attention. The science is almost certain to continue to progress rapidly, enabling us to predict human traits with increasing accuracy and raising serious questions which in our view need equally serious discussion.

## 2. Genomic research and social policy

Harden & Koellinger [7] have argued that: ‘The genetic revolution will change our lives and our societies whether or not we want it to’ [7, p. 574]) and that it is therefore vital that genomic research is taken seriously. They make a case that success in life is a genetic lottery (see [8] for a fuller exposition of this idea) and that this raises important policy questions about fairness and the distribution of resources. In support of their argument, they cite studies reporting associations between polygenic scores (clusters of genetic variants associated with a particular trait or outcome) and phenomena such as social mobility, achievement of developmental milestones, reading proficiency [9] and personal wealth [10]. They also explain how genomic research can help us to test individual differences in response to a range of interventions. For example, Barcellos, Carvalho & Turley [11] found that an additional year of compulsory schooling for all young people in the UK was associated with improved obesity-related health outcomes and lung function when those young people reached middle age, and that these effects were most positive for those with a higher number of genetic risk factors for a high BMI.

Findings such as this provide cause for optimism that behavioural genomic research may be able to offer policy-makers a new ‘genetic lens’ [12] through which to view society and provide information that could make a useful contribution to evidence-based decision-making. Asbury & Wai [12] have made a case that it is important to communicate findings from behavioural genomics to policy-makers so that they have access to this robust and reliable body of evidence and can take it into account in their decision-making. However, they also argue that no necessary policies follow from genetic research and that it is important to be mindful that the findings raise at least as many questions as they offer solutions.

In response to this paper [12], while agreeing that a cautious approach is advisable, another group of researchers [13] did propose a specific policy that follows directly on from genetic research into reading development and reading difficulties. There is good evidence that differences between children in their reading proficiency are primarily explained by genetic differences between them and by experiences that are not shared by two children raised in the same family (e.g. [14]). As a result, Byrne *et al*. [13] proposed a policy in which funding available for pupils struggling with learning to read should be allocated to the individual learner rather than to their school. This policy suggestion is rooted in genetic research but does not require any genetic screening, just the observation that a child is struggling with reading. They make a case that intervention could take the form of paying for an additional teacher or teaching assistant in order to allow the class teacher to spend time supporting the specific child to whom the funds were allocated. Alternatively, a vouchers mechanism could be used, allowing parents to access specialist support outside of school. In this case, robust and reliable evidence from behavioural genetics can be used to show how funding targeted at an individual is likely to be more effective than funding targeted at an institution (the more common approach) because individual differences are heritable. The authors also emphasize the importance of evaluating the effectiveness of their proposed approach.

It is clear that researchers are beginning to think about the implications of genomic research for social policy, but that this research is in its infancy. The field is urging caution, but there is also a small amount of evidence of cautious optimism.

## 3. Early Intervention Foundation report

The EIF is a UK government What Works Centre dedicated to ensuring that effective early intervention is available to support children and young people at risk of poor outcomes. As a knowledge broker, the EIF sits at the interface between research and policy, generating and mobilizing evidence to influence national and local policy as well as practice.

In 2021, the EIF published *Genetics and early intervention: exploring ethical and policy questions* [5]. This report was motivated by the rapid advances in genomics which suggest that it may become increasingly possible to identify—from birth—children with increased odds of experiencing outcomes such as struggling at school or being diagnosed with a learning, behaviour or mental health condition. While such predictions are far from perfect, they do appear to be meaningful at the group level like other important predictors such as eligibility for free school meals in the UK. Since intervening early relies on identifying children at risk of experiencing challenges as they grow up, there is a prima facie reason to think that genetic data could potentially be combined with demographic and diagnostic data to better target services. However, incorporating DNA data into early intervention policy or practice raises some profound ethical questions. It is also important to consider whether genetic data requires different considerations and regulations than other types of data, and whether it is ethical to target policies on the basis of risk rather than diagnosis.

The EIF's guiding question was: can genetic data be used to improve outcomes for children and families without marginalizing individuals, entrenching disadvantage or increasing inequalities? To explore these issues, a series of workshops were convened with experts from a wide range of backgrounds. The workshops identified three key themes:

1. *Enhancing public and policy-maker understanding of genetic research is a key priority.* There was broad agreement among workshop participants that limited knowledge is one of the major barriers to considering the application of genetic research in social policy and that a programme of well-designed and accessible public communication is needed to address low knowledge, poor understanding, misconceptions and low trust.
2. *Our current understanding is based on samples largely from people of European ancestry, and this is a problem for science and society.* There was a high level of consensus that diversification of the research community, and a proactive, genuine and meaningful approach to the co-construction of research with the communities affected by it, are prerequisites for progress in this area. There was unanimous agreement from participants that any application of polygenic scores in the context of social policy will be premature so long as this diversity deficit persists.
3. *DNA data can help researchers to better understand development and identify what works to support those at risk of poor outcomes.* This was an area of strong consensus and was viewed as less ethically challenging than other areas discussed. Participants argued that collecting genetic data as a routine part of longitudinal cohort studies in order to better understand the interplay between genetics and the environment represents a particularly promising area for progress. Another was improving the precision of estimates from randomized control trials and other impact evaluations by including genetic data alongside socioeconomic or psychosocial indicators to help understand what works for whom.

The EIF workshops also explored two hypothetical scenarios, one in which polygenic scores were used to identify those at risk of poor educational attainment; the other in which polygenic scores were screened to identify those with an increased probability of experiencing developmental conditions such as autism, ADHD or dyslexia.

On the issue of whether genetic information could be used to channel additional resources to pupils identified as being at elevated risk of doing poorly in school—much as we do in England where schools receive the pupil premium for children who are eligible for free school meals as well as those who have been looked after by the state—opinion was divided. While there was some cautious support for the argument that this could help to support more children and young people at risk of performing poorly, the overall feeling was that the risks of using DNA information to shape school funding currently outweigh any potential benefits, and that there are more pressing matters that should be addressed to improve educational outcomes.

By contrast, the majority of participants believed that polygenic scores could be useful in the future to identify those with an elevated probability of being affected by developmental conditions, while also acknowledging that this would present a range of ethical issues that would need to be addressed first. The prevailing view of workshop participants was that the optimal approach to using these scores would be for monitoring those identified as being at risk rather than automatically intervening, as we do with breast cancer for instance.

The EIF report made a series of specific recommendations for the UK government and the international research community. These were focused on establishing an independent body to advise the government on the implications of using genetic data in social policy-making; launching a comprehensive programme of public awareness-raising; prioritizing ancestral diversity in biobanks via a carefully designed participatory research approach; and routinely incorporating DNA data collection in longitudinal and evaluation studies.

The EIF report was intended to begin an important conversation. The authors recommended getting ahead of the science to explore whether genetic information could and should be used in the design and delivery of early intervention and social policy before such decisions become pressing. Similar concerns seem to sit behind other explorations of the role of genetics in informing social policy, including the report from the UK GO Science.

## 4. UK Government Office for Science report

In January 2022, GO Science published *Genomics beyond health: what could genomics mean for wider government?* [6]. GO Science advises the Prime Minister and members of the Cabinet to ensure that government policies and decisions are informed by the best scientific evidence and strategic long-term thinking, and is led by the Government Chief Scientific Officer, currently Sir Patrick Vallance. In his preface to the report, Sir Patrick stated that:

While genomics has the capacity to be transformative for many aspects of society, there are ethical, data and security risks that may accompany its use … As the power of genomics increases, and uses proliferate, government will increasingly face choices about whether this approach still ensures the safe integration of genomics into society. Capitalising on the UK's various strengths in genomic infrastructure and research will require a coordinated effort across government and industry. [6, p. 3]

This wide-ranging report explains genomics to an audience of non-specialists and considers the implications of progress in genomics for Whitehall departments. The authors explore ‘what is possible now, what might be possible in the future, and … where the capabilities of genomics are potentially being oversold’ [6, p. 6]. They look at how the science of genomics might be used in areas such as employment, education and criminal justice.

In relation to education, the authors noted high heritability estimates for educational attainment and the fact that education represents one of the most heavily studied clusters of traits in genomics outside of medicine. They also explained that while current predictions of academic outcomes at age 16 from polygenic scores are broadly equivalent to prediction from measures of socioeconomic background, they provide no added benefit over and above measures of prior attainment. However, since genetic data can be measured at birth it may provide a means to offer earlier and more targeted support to individuals, and improvements in our understanding of genetic influences on attainment could support better understanding of the role the environment plays in influencing outcomes.

The report notes that, while there is no use of genomic testing or genomic information in the UK education system, currently there is no UK regulation on the use of genomics in education and little to stop direct-to-consumer (DTC) testing providers offering tests of educationally relevant traits, despite very low predictive power at an individual level. The authors also discuss some of the ethical challenges that exist around using genetic data in the education system, including the fact that current polygenic scores are more accurate for individuals of European ancestry due to the fact that the samples these measures were produced from were overwhelmingly of European descent. Also, if parents have their child tested this could potentially undermine the child's autonomy and right to an ‘open future’ and could lead to stigmatization, discrimination and feelings of fatalism for that individual, all points that were also raised in the EIF consultation.

The report goes on to discuss data, security and public attitudes to genomics. This includes the fact that genetic data is highly valuable information for the individual and their immediate family and therefore needs a high level of protection from unintentional disclosure and a purpose-built legal framework for genomic databases. It also highlights key issues and risks for policy-makers and decision-makers to consider, including the need for guidance on when genetic tests should be used outside of the health sector, and how results should be interpreted as the predictive power of such tests grows. It recommends a systematic assessment of how genomic data could be used on a sector-by-sector basis, along with appropriate regulations around genome-based discrimination. Greater regulation of DTC genetic testing companies is also recommended.

## 5. Areas of consensus between the two reports

Both reports agreed on two key areas as the basis for their policy recommendations. The first of these relates to public knowledge, understanding and perceptions of genomic research, with the EIF report advising the government to launch a comprehensive programme of public awareness-raising and discussion, and the GO Science report recommending greater public dialogue on the current and future uses of genomics. The second area is equality, diversity and inclusion and, more specifically, ensuring that large genomic databases such as biobanks are representative in terms of ancestry. Both reports urged those in charge of such datasets to work towards genuine representativeness and were clear that, in the absence of representative datasets, any policy-making would be inappropriate as it would risk entrenching existing disadvantages.

Beyond these shared priority areas, the GO Science report's recommendations were focused primarily on the need for regulation around the use of genomic data in social policy and around the DTC genetic testing market. It also made a case for the potential benefits of a unified legislative framework for genomic databases. The focus of the EIF report was more on activities to guide policy, such as establishing an independent body to advise the government on the ethics of using genetic data in social policy and advice to the parliamentary Science and Technology Committee to hold an inquiry into the implications of genetic research for UK social policy. The EIF report also presented recommendations for research funders, including advice to increase the focus on genomic data in projects where it could add value, particularly intervention studies. In the next section, we review literature related to the two areas in which both reports made clear recommendations.

## 6. A review of literature relating to the reports' key findings and recommendations

### 6.1. Public knowledge and understanding

Both reports agreed on the importance of public dialogue regarding how progress in genomic science is applied to social issues. However, the research available suggests that public knowledge and understanding are generally low. It seems unlikely that much constructive large-scale dialogue can take place if the public are not sufficiently genetically literate to engage meaningfully in it. Such findings support the call in the EIF report for a government-led public information programme, something that other researchers have also called for [15,16].

Studies have found that genetic literacy is lower than would be necessary as the baseline for meaningful public dialogue, even in highly educated populations. For example, in Chapman *et al*.'s [16] international sample the average score on an 18-item test of genetic knowledge was 65.5%, in spite of 89.6% of the sample being educated to degree level or higher. The test covered basic functional genetic literacy (e.g. What is a genome?; What is the main function of all genes?) and a small number of items asking whether the genetic contribution to risk of developing conditions such as schizophrenia and autism comes from one gene or many genes. This sample showed very limited understanding of polygenicity—the finding that most aspects of human health and behaviour are influenced by many genetic variants of individually small effect—and that these variants interact with each other and with the environment. Rather, 30% believed, falsely, that conditions such as schizophrenia and autism are caused by a single gene. In Jordan, Almomani *et al*. [15] found that 43.4% of their sample had relatively good knowledge of genetics at a very basic and heredity-focused level. Again, over half of the sample were educated to degree level.

Using their Public Understanding of modern Genetics and Genomics Scale Carver *et al*. [17] observed that young adults' knowledge and understanding was particularly low on mechanisms for genetic expression and genotype-environment interplay. Condit [18] found a similar pattern of reasonably good knowledge of core genetic concepts (e.g. heredity as widely taught in school science curricula) but not of genotype–environment interplay, mechanisms for expression of genetic risk or the idea of probable risk, which is central to understanding the predictive role of new tools such as polygenic scores. In relation to this, Agurs-Collins *et al*. [19] pointed out that poor numeracy skills may be as much of a problem for social engagement with developments in genomic research as poor genetic literacy. For instance, the results of DTC genetic tests are usually reported as risk ratios, without access to expert guidance or genetic counselling. Low genetic literacy and poor statistical understanding can lead to misconceptions and anxiety in these circumstances.

Other studies of genetic literacy have focused on particular groups of professionals and have, for example, found that knowledge is low among teachers [20,21] and medical professionals [22]. Importantly, there is also evidence that openness to learning more about genetics is high among teachers [20]. Gallop *et al*. [22] point out that many people fail to recognize the limits of their knowledge, leading to misplaced confidence. It is easy to see how this low-knowledge landscape could act as a hotbed for a public dialogue that is beset by misconceptions, and this is clearly a problem to be avoided.

The need for genetic literacy education as a precursor to public dialogue seems clear. The key points to convey include the knowledge that variation in all characteristics is influenced by genes; that most characteristics are influenced by dozens or even hundreds or thousands of genetic variants; that genes interact with the environment in complex ways; and that genetic effects are not deterministic. A well-designed public education programme is a priority as this science is progressing rapidly, and decisions about whether and how its findings should be used in a social policy context must be made by as wide and representative an array of stakeholders as possible. So, given that education is needed across ages and educational backgrounds, where and how could and should this education optimally take place?

Most people receive the majority of their education about genetics from school-based Biology lessons. However, these lessons tend to focus on Mendelian inheritance and transmission genetics [17]. Where we see pockets of relatively good genetic literacy it is in this area. Carver *et al*. [17] have therefore called for a greater emphasis on modern genetics and genomics in the science curriculum, to reflect the state of the science today. This call was made 5 years ago and, to our knowledge, has not yet been responded to in a meaningful way. The current educational emphasis on heredity rather than heritability may explain why some studies have identified good knowledge of core concepts alongside poor knowledge of polygenicity, mechanisms for gene expression and—perhaps most importantly—genotype–environment interplay. Carver *et al*. [17] argue that the current emphasis of Biology education encourages young people to think deterministically. It fails to acknowledge that the intergenerational transmission of heart disease, diabetes, cognitive ability and personality traits differs from the intergenerational transmission of form and colour in peapods. It therefore seems fundamentally important that we work towards introducing behavioural genomics alongside Mendelian genetics in school Biology curricula.

Donovan *et al*. [23] have also argued that traditional genetics education encourages essentialist beliefs such as the belief that racial groups differ, in terms of their cognitive ability and behaviour, because of group-specific genetic make-up. They developed and evaluated an intervention designed to enhance understanding of human genetic variation and race and assessed how it interacted with genetic literacy to affect essentialist thinking. They found that, after the intervention, those with good genetic literacy showed greater reductions in essentialist thinking than those with poor genetic literacy. Such findings support the need for enhancing genetic literacy and including up-to-date information on population genetics, genotype–environment interplay and polygenic risk. For instance, teaching pupils about the distinction between socially constructed concepts of race and ethnicity and more biologically relevant constructs such as ancestry could reduce misconceptions that can lead to racism. Researchers such as Stern & Kampourakis [24] have also called for this change in science education, arguing that common misunderstandings are the result of a deficient genetics curriculum. We argue that this deficiency is a tangible issue that education policy-makers could address with a tangible solution.

There is also evidence that many people gain their knowledge and understanding of genetics and genomics from the media. For example, Agurs-Collins *et al*. [19] found that of the 36% of their US sample who were aware of DTC genetic testing, the majority cited radio, TV and the Internet as the sources of their awareness. Almomani *et al*. [15] also found that almost half of their approximately 5000 participants gained their knowledge of genetics through the media. Gallop *et al*. [22] argue that although public interest in genetics is high—clearly a good thing for the public dialogue called for in these reports and by researchers—it is important to note that people tend to access information passively, and to absorb messages that align with their intuitions. When the media presents simplified explanations, focusing on something ‘being genetic’ rather than on the complex genotype–environment interplay that is almost always involved, this may hit home as it aligns with widespread essentialist beliefs [25] and, in turn, may foster further misconceptions. Deficiencies in how science is communicated in the media have also been identified as promoting essentialist thinking and, in doing so, potentially biasing the public against policies designed to tackle inequality [26,27]. This is important from a social policy perspective, particularly in light of the far-right thinking that has been rising around the world in recent years. This suggests that any public education programme will need to recognize, and indeed harness, the power of the media but also work hard to make complex messages accessible.

It is interesting and important to note that Almomani *et al*. [15] found, in their large Jordanian sample, that more knowledgeable participants had more concerns about the uses of genomic science than those who were less knowledgeable. Chapman *et al*. [16] also noted that greater genetic knowledge was associated with less deterministic views. This type of informed critical thinking is essential to the quality of the public dialogue that both EIF and GO Science have called for. To facilitate it, we first need to proactively build public knowledge and work to ensure that this knowledge is understood by the broad sweep of society. This is likely to involve both a change in school-based Biology education and a media-focused programme of education and engagement. As others have also argued (e.g. [16]), genetically literate societies are required for constructive public dialogue on vitally important issues including gene editing, prenatal screening and attempts to use probabilistic information as a basis for intervention.

That said, it is important to consider what should happen if we fail to develop a genetically literate society in the coming years or decades. We would argue that society has a responsibility to not allow this to happen. If we do not put in place mechanisms for children and young people to understand the way in which the genome relates to individual differences in behaviour, and the implications of that, we are reducing their capacity to take part in democratic decision-making around the kind of society we want to be. The fact that we do see evidence of genetic literacy around Mendelian concepts suggests that an equivalent well-designed curriculum focused on more modern understandings of genomics has a good chance of achieving a similar effect.

### 6.2. Diversifying genomic datasets

It is acknowledged both within and without the scientific community that the pre-eminence of participants of European ancestry in biobanks and GWAS datasets is a problem in urgent need of a solution [28]. This was also acknowledged loudly and clearly in the EIF and GO Science reports, both of which concluded that incorporating DNA data into social policy-making is out of the question for as long as this diversity deficit persists.

Gurdasani *et al*. [28] pointed out that although diversity in genomic datasets had been gradually increasing over the previous 5 years, it was still the case that 78% of participants were of European genetic ancestry, with 9% of East Asian ancestry and all other populations making up only 13% of the data. This is a substantial problem because, while many common variants are shared across ancestries (because they pre-date migration out of Africa), there are also important differences due to factors including genetic drift and natural selection. We know that polygenic scores that are developed from European-only summary statistics—a common situation—perform poorly when applied to non-European populations [29]. This means that incorporating such scores into policy or practice in education and social support would be highly likely (if the prediction proved beneficial, on average) to exacerbate existing inequalities. This is the reason that both reports agreed that such a step should not be considered until this problem has been solved.

The genomics research community is acutely aware of the issue and is taking steps to address it via initiatives such as the Pan-UK biobank [30] and NIH's TOPMed [31]. This is vital to the development of fair and effective preventive, therapeutic and diagnostic strategies [28]. However, it is not an easy problem to address and will require a great deal of resource to be invested in building partnerships with a wide range of communities and governments, and developing a sustainable infrastructure that can support collaborative research. Both reports fully endorse the need to slow down and address these challenges so that the world can move forward together in harnessing the potential of genomic research for social policy (if it is deemed wise to do so).

While this effort unfolds, a clear case can be made for using the data we already have. There are indications that the 13% of DNA data gathered from participants of non-European and non-East Asian ancestries accounts for 38% of the significant associations in the GWAS catalogue [30]. For example, individuals with African and Latin American genetic ancestry appear to contribute disproportionately to discovery, in spite of smaller samples [32]. Researchers have been encouraged to resist the pull of the European ancestry sample size—a significant draw when seeking variants of very small effect associated with specific outcomes—to also conduct trans-ethnic and ancestry-stratified analysis in the smaller available samples of participants with substantial non-European genetic ancestry [30].

In short, it is clear that significant steps are being taken to diversify genomic datasets and commentary in the literature appears to support the two recent reports' concerns that using DNA-based variables in a social policy context will be premature until these steps bear fruit. The success of the endeavour will be highly dependent on genuine and far-reaching co-production of genomic research with the communities involved.

## 7. Conclusion and future directions

Our main conclusion is that it is positive that in-depth thinking about the implications of genomic research for policy is taking place at the heart of the UK government and in government-linked organizations such as the EIF. The recommendations on which the EIF and GO Science reports overlapped—those related to public education and engagement and to diversifying genomic databases—are quite telling. Both represent activities that are essential precursors to any development of genetically informed social policy. It might be that the conduct and outcomes of these endeavours lead to genetically informed social policy-making or, alternatively, a decision to prevent the approach. Either way, such decisions should not be taken without these programmes of public education and diversification being put in place, as they are fundamental to the decision-making process. We would like to add the caveat that this paper has drawn primarily on research conducted in the global north and has largely focused on social policy in the same geographical area, with a specific focus on the UK. Consideration of the importance of genomic research for social policy in the global south lies beyond the scope of the current paper but represents an important topic for future discussion and development.

In our view, the scientific community is handling the important issue of diversification. We would urge scientists to ensure that a genuinely collaborative and participatory approach is taken, as this is likely to be essential to the success of the endeavour. However, there is little that policy-makers can currently do in this space beyond encouraging and, where needed, supporting the process. For example, research council funding could make diverse sampling a requirement for relevant funded projects.

By contrast, there is a strong role for government and policy-makers to play in relation to public education. In this case, a review of the ways in which genetics is currently taught in secondary schools (Key Stages 3–5) is strongly indicated, with a view to finding space for a unit introducing behavioural genomics and comparing it with Mendelian genetics. This is likely to represent a substantial component of a sustainable solution to the low genetic literacy problem that we see. It will also be important to develop a public education programme for society at large, disseminated through multiple media channels to reach a diverse audience. If policy-makers want to prepare for a future in which DNA data is widely accessible—what Harden & Koellinger [7] referred to as ‘the genetic revolution’ [7, p. 574]—then public education, including the school Biology curriculum, is the place to start. Policy-makers need to prepare for this genetic revolution by making sure that they themselves, as well as society at large, have sufficient knowledge and skills to be able to address the profound ethical challenges raised by the possible incorporation of DNA data into social policy.

## Data Availability

This article has no additional data.
